# Supplementary material for: Heterogeneous effects of battery storage deployment strategies on decarbonization of provincial power systems in China
Source: Nat Commun. 2023 Aug 11;14:4858. doi: 10.1038/s41467-023-40337-3 (PMC10421958; doi:10.1038/s41467-023-40337-3)
Supplement: Supplementary file 1 — Supplementary Information [file 41467_2023_40337_MOESM1_ESM.pdf]

Supplementary Information for:

Heterogeneous effects of battery storage deployment strategies on decarbonization of provincial power systems in China

Liqun Peng[1], Denise L. Mauzerall[1,2*], Yaofeng D. Zhong[3], Gang He[4,5*]

[1]Princeton School of Public and International Affairs, Princeton University, Princeton, NJ 08544, USA
[2]Department of Civil and Environmental Engineering, Princeton University, Princeton, NJ 08544, USA
[3]Department of Mechanical and Aerospace Engineering, Princeton University, Princeton, NJ 08544, USA
[4]Department of Technology and Society, College of Engineering and Applied Sciences, Stony Brook University, Stony Brook, NY 11794, USA
[5]Marxe School of Public and International Affairs, Baruch College, City University of New York, NY 10010, USA

*Correspondence:
mauzerall@princeton.edu
gang.he@stonybrook.edu

Supplementary Note 1: Electricity demand projections

The total electricity demand projection is derived from below two-degree scenario of China National Renewable Energy Outlook 2020 which assumes an electrification of an increasing portion of the economy. We allocate total electricity demand into provinces based on Hu's presentation. The total electricity demand projection is derived from below two-degree scenario of China National Renewable Energy Outlook 2020. We allocate total electricity demand into provinces based on Hu's presentation (Supplementary Fig.1). In 2050, Hebei, Shandong, Jiangsu, and Guangdong provinces have the highest electricity demand. Next, we gather the monthly and daily profile of power load by province and estimate the hourly electricity demand by province.

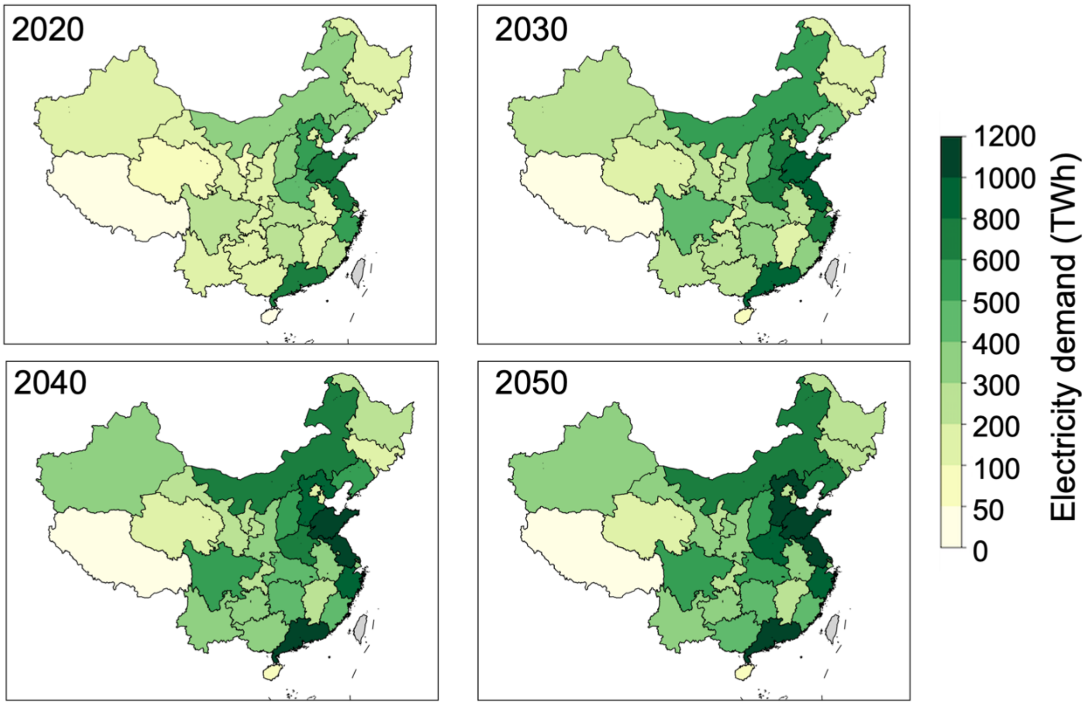

Supplementary Fig. 1. Distribution of electricity demand by province from 2020-2050. The map of China is sourced from the National Catalogue Service for Geographic Information[1].

Supplementary Note 2: Cost information

Capital costs are amortized over the expected lifetime of each generator or transmission line. Only those payments that occur during the period covered by the study are included in the SWITCH-China objective function. We set up three cost trajectories for solar photovoltaic (PV), wind, and battery storage. Technology adoption, learning-by-doing, economies of scale, and manufacturing localization are driving the cost decease of solar PV, wind, and battery storage.

Supplementary Fig. 3, 4 and 5 show the capital costs assumptions of renewables and storage. The operation and maintenance (O&M) costs for each of these three technologies in our model are estimated to be 1% of their respective capital costs under the given scenario. Charging and discharging batteries results in a "roundtrip" loss of energy. In our model, we assume a roundtrip energy loss of 12%, 15% and 20% for 1h batteries, 4h batteries and 10h batteries during battery charge and discharge[2]. The hourly based wind and solar capacity factors used in this study are estimated using the model in He et al.[3]

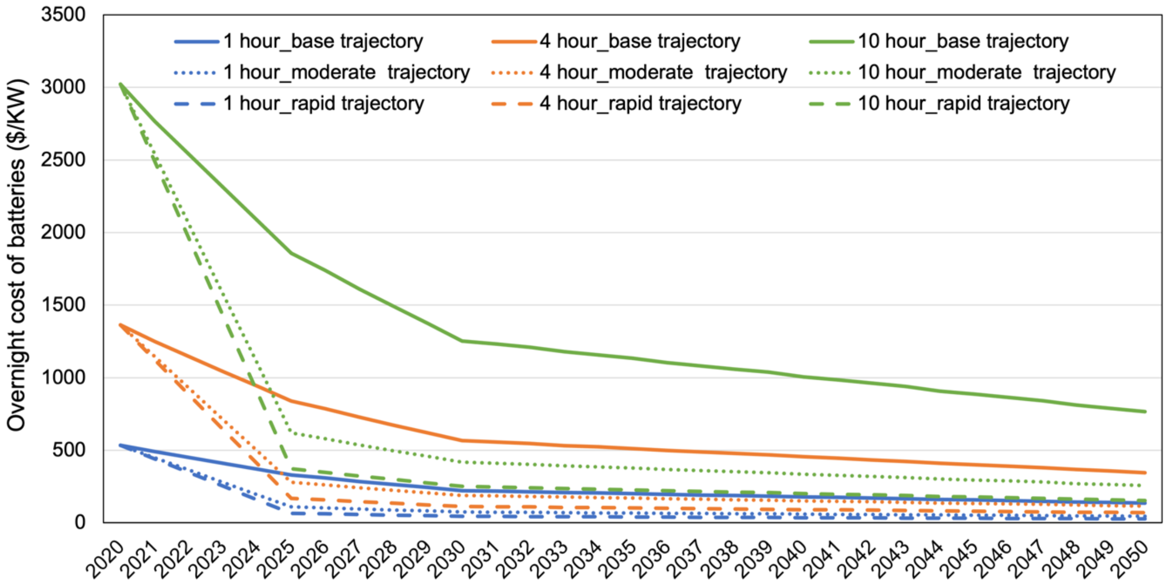

Supplementary Fig. 2. Capital cost of batteries in the model.

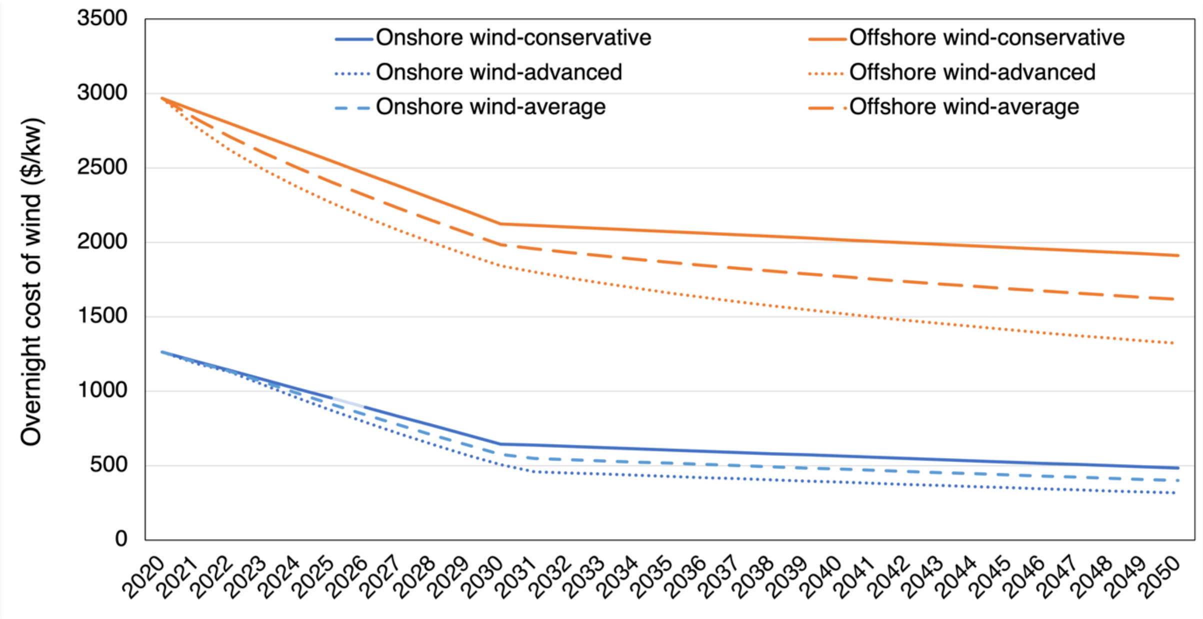

Supplementary Fig. 3. Capital cost of solar PV in the model.

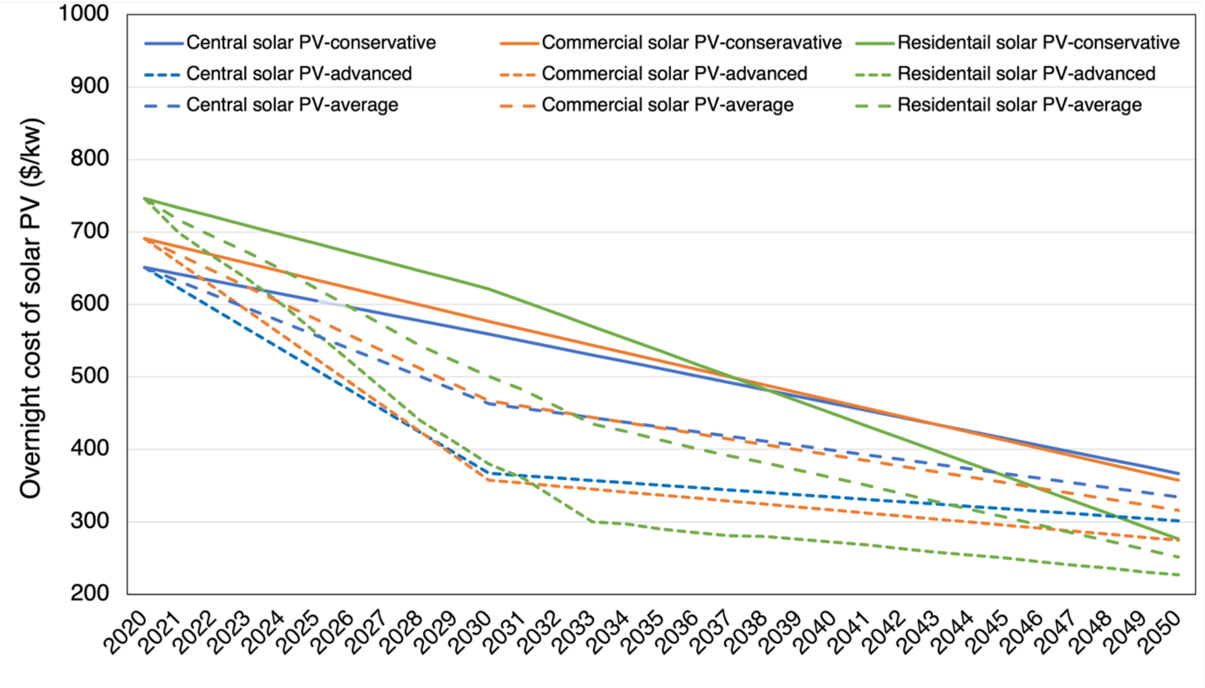

Supplementary Fig. 4. Capital cost of wind in the model.

Supplementary Note 3: High carbon prices can drive decarbonization of China's power system.

Decarbonization of the power system can be driven by regulatory actions (e.g., requiring the closure of old inefficient power plants) or by market mechanisms like emission trading and carbon prices. In our model, the decarbonization rates are driven by carbon prices. Figure 5 presents the effect of low and high carbon prices that both increase over time on annual CO_2 emissions, optimal power capacity and generation, battery storage and system costs. The optimal system is one which minimizes total costs. Total costs equal the physical system costs plus CO_2 emissions weighted by carbon price.

Here, we highlight several observations from Supplementary Fig. 5. First, we find that in each year, higher carbon prices indeed result in higher penetration of renewables (rapid decarbonization rates) than lower carbon prices with particularly rapid growth in solar generation because the capital costs of solar are lower than those of wind (Supplementary Fig. 5a and 5b). To balance the increased variable renewable generation, more battery storage must be installed (Supplementary Fig. 5e) thus leading to greater battery storage deployment under high and rapidly increasing carbon prices. Second, Supplementary Fig. 5e shows that for both low and high carbon prices, the deployment of 4-hour battery storage is favored over 1-hour and 10-hour battery storage. This is because long duration energy storage is needed to better utilize intermittent renewable energy, but storage in 10-hour batteries is more costly than in 4-hour batteries per kW. Nevertheless, the capacity of long duration battery storage (10-hour) increases more when carbon prices are high than when they are low, except in 2050. Third, high carbon prices will accelerate the decarbonization of the power system (Supplementary Fig. 5a and 5b) while only marginally increasing system costs (Supplementary Fig. 5f). In addition, we find that nuclear power plays an important role in China's power system, especially when the penetration of renewables is high. Nuclear generators produce a consistent and reliable output, offering baseload power to stabilize the grid and along with batteries mitigate the intermittency of renewable energy sources.

However, battery storage plays an increasingly important role over time as renewable generation increases (Supplementary Fig. 7 and 8) and CO_2 prices increase (Supplementary Fig 5a). In 2030, few batteries are available to store excess solar and wind electricity. By 2050 grid-connected batteries store around half the power generated by renewables and discharge the stored power to the grid primarily during the evening peak period (after 4pm) as shown in Figure 6.

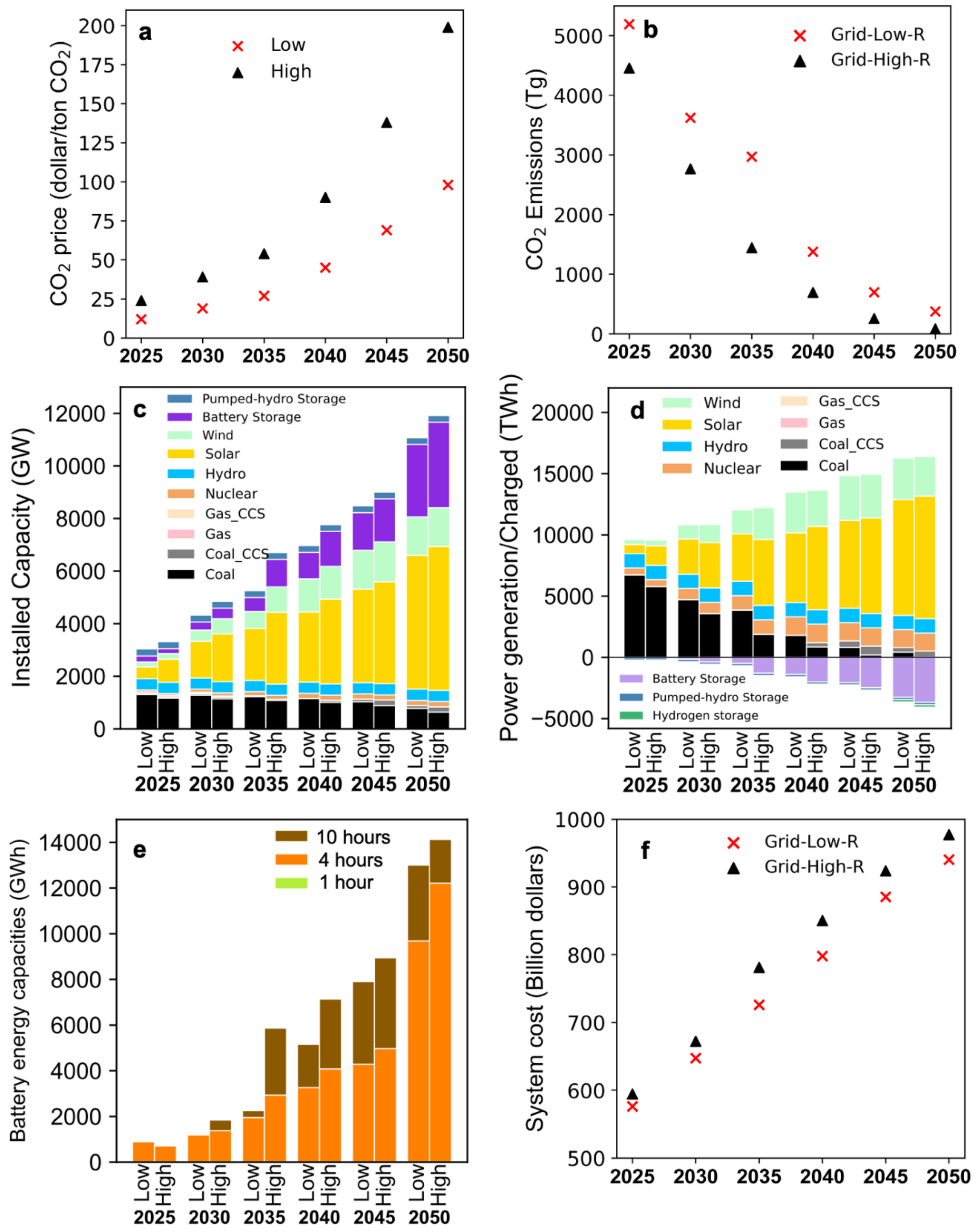

Supplementary Fig. 5. Implications of high and low carbon prices on the national power system. **a** CO_2 prices over time, **b** optimal national CO_2 emissions, **c** optimal installed capacity of each generation technology, **d** optimal power generation, **e** optimal installed battery energy capacities by storage duration (battery storage capacities of 1-hour storage duration are tiny compared to longer storage durations), and **f** optimal system cost of the Grid-connected battery strategy with rapid battery cost decreases. Low and High in **a, c, d,** and **e** represent low and high carbon prices. Grid-Low-R/Grid-High-R indicates batteries which are connected to the grid that have low or high carbon prices and rapid decreases in battery costs. Coal_CCS and Gas_CCS represent coal and gas power generation with carbon capture and storage.

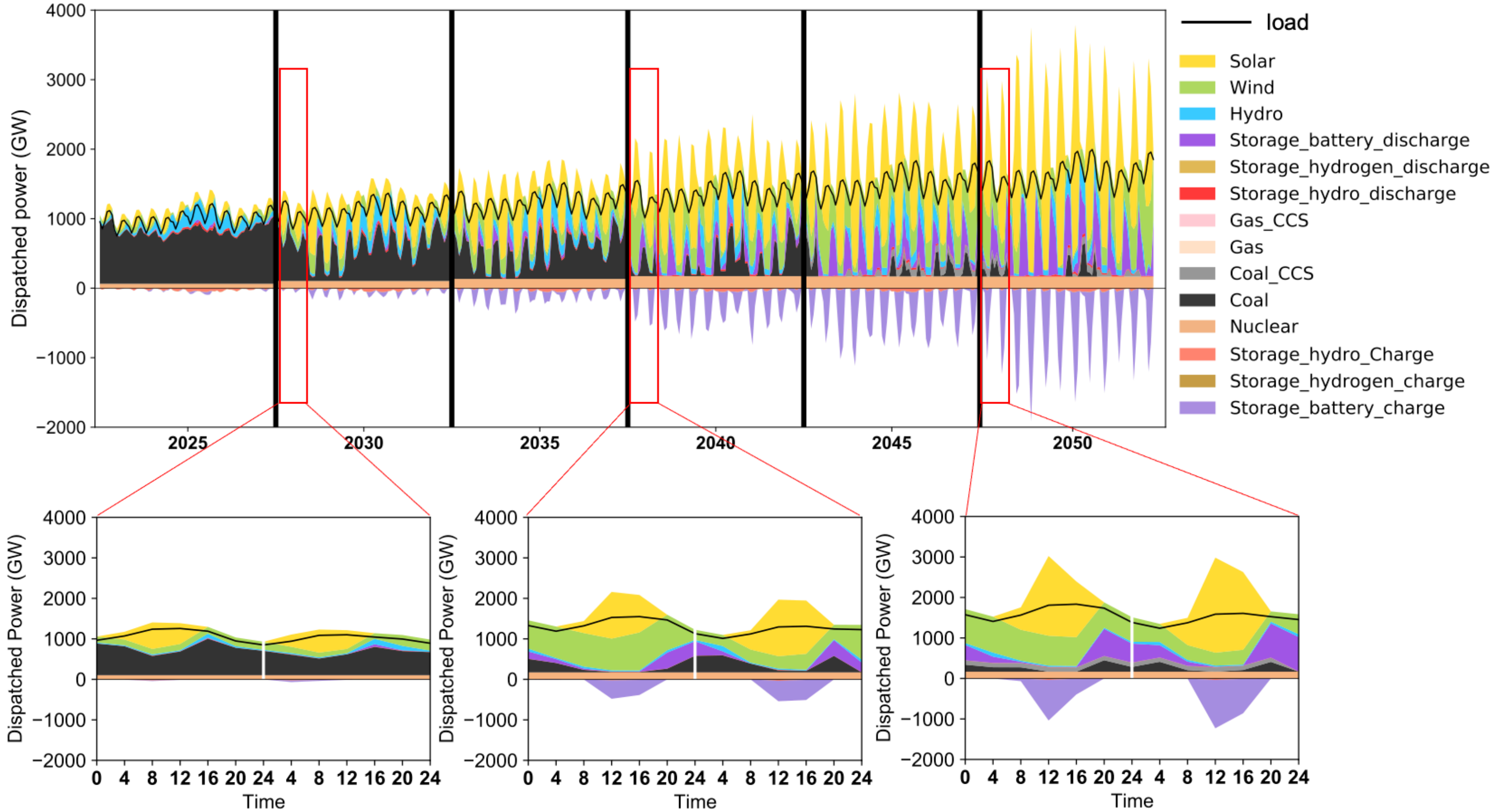

Supplementary Fig. 6. Dispatched power and load profiles from 2025 to 2050 under Grid-Low-R scenario (Grid connected batteries with low carbon prices and rapid decrease in battery storage costs). Coal_CCS and Gas_CCS represent coal and gas power generation with carbon capture and storage.

Supplementary Note 4: The effect of battery storage cost reduction trajectories on national installed battery storage capacity and CO_2 emissions

At each time step, the evolution of battery costs has significant impacts on installed capacity of battery storage, CO_2 emissions, and electricity costs. Other than the three cost trajectories specified in our scenarios (Base case, Moderate decrease of battery storage costs and Rapid decrease of battery storage costs), we also examine an extreme no-storage scenario where we assume the cost of battery storage is prohibitively expensive, and thus no battery storage is built in the power system. From Supplementary Fig. 7, we observe that both CO_2 emissions and electricity costs under the no-battery storage scenario are the highest of all battery storage scenarios. The cost trajectories with the lowest prices (R) results in the highest installed capacity of battery storage, lowest CO_2 emissions, electricity costs and vice versa under both low and high carbon prices. This indicates that success in decreasing battery production costs will have valuable benefits for both CO_2 mitigation and electricity cost reductions. We conclude that the cost trajectories with the lowest (rapid cost decrease) storage price along with high carbon prices achieve the lowest CO_2 emissions across all eight scenarios shown in Supplementary Fig. 7.

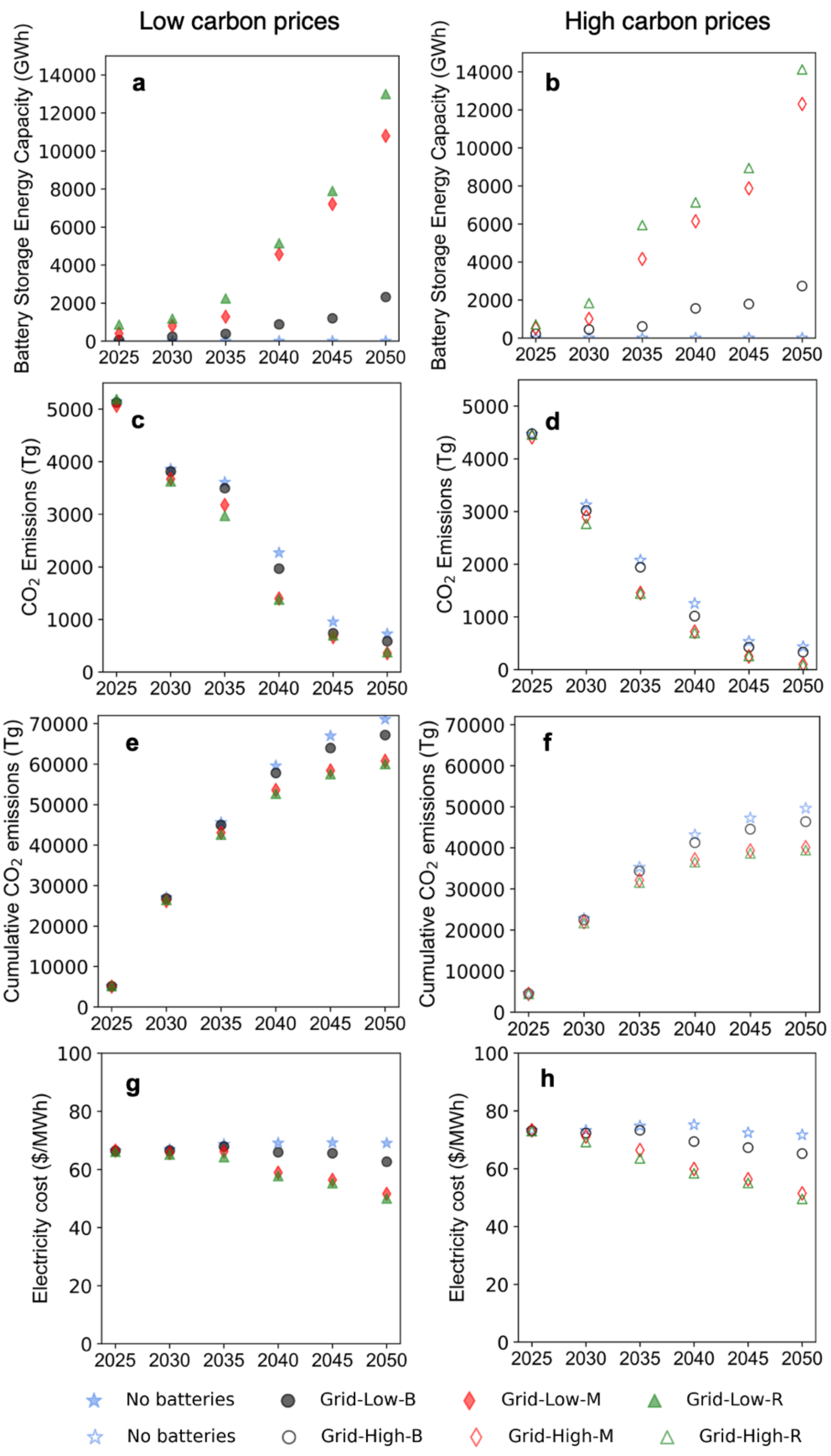

Supplementary Fig. 7. Implications of low and high carbon prices and battery storage cost reductions on the national power system. Under low (left column) and high (right column) carbon prices we show temporal pathways of **a** and **b** optimal battery storage energy capacity, **c** and **d** optimal CO_2 emissions, **e** and **f** optimal cumulative CO_2 emissions, and **g** and **h** optimal electricity costs under Grid-connected battery strategy and low(high) carbon prices with no batteries, base case (B, high battery costs), moderate (M, medium battery costs) and rapid (R, low battery costs).

Supplementary Note 5: Sensitivity analysis of demand loads on battery storage deployment

To evaluate how demand load affects the battery storage deployment, we conduct sensitivity analysis of installed battery capacity under various demand loads. We refer to the projected demand (Supplementary Note 2) and demand profile derived from 2015 as the business as usual (BAU) scenario, then we set up four scenarios - 60% 80%, 120% and 140% of projected demand (60% BAU, 80% BAU, 120% BAU and 140% BAU), to explore the impacts of demand loads on battery storage deployment and the differences in the impacts under four different battery deployment strategies.

We find that, in general, battery deployment has a positive relationship with demand load, as shown in Supplementary Fig. 8. Higher demand load results in larger battery storage deployment. However, the correlation between battery storage deployment and demand load is not linear. This is because changes in demand load will further impact the supply and demand balance in each province, which make the situation more complicated. It's valuable to explore this further in future studies.

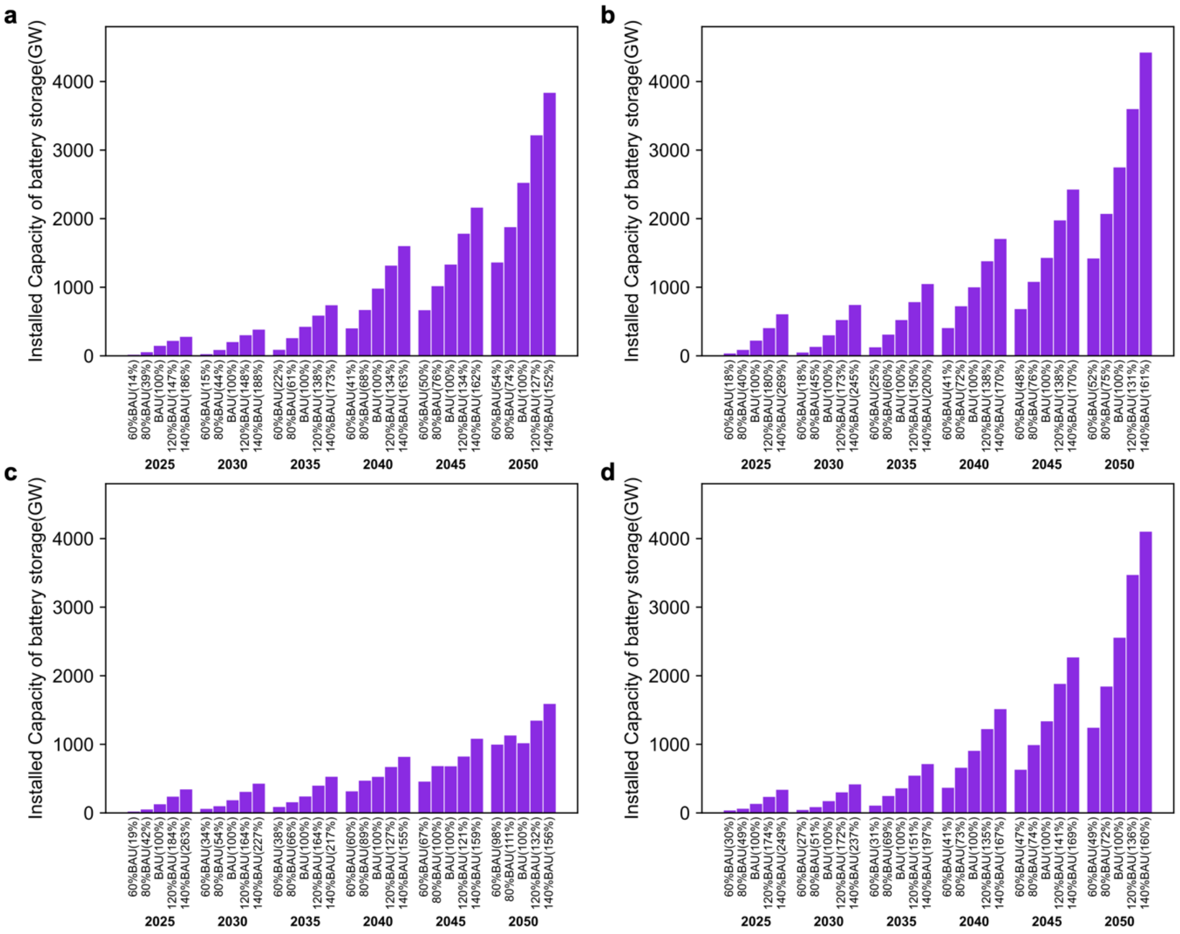

Supplementary Fig. 8. Sensitivity analysis of installed battery capacity from 2025-2050 under business as usual (BAU), 60%, 80%, 120%, and 140% of BAU demand for **a** RE-connected, **b** Grid-connected, **c** Demand-side, and **d** Mixed battery deployment strategies with low carbon prices and rapid decreases in battery costs. Percentages in the brackets show the ratio of installed battery storage in each demand scenario compared to the BAU demand.

Supplementary Note 6: Increase carbon prices to achieve the lowest CO_2 emissions in the Mixed battery strategy.

To properly capture the goal of carbon emissions mitigation, we can increase the weight of CO_2 emission in our objective function. Here, we test the comparison results among four battery strategies with the increase of carbon prices. First, we test the impact of carbon prices that increase to 100 times of our low carbon price scenario. In this case, we find that the Mixed strategy achieves both the lowest total cost and CO_2 emissions out of the four battery strategies (RE-connected, Grid-connected, Demand-side and the Mixed) (Supplementary Table 1). Next, we investigate the threshold carbon prices that enable the Mixed strategy to achieve both the lowest total cost and CO_2 emissions. We find that when carbon prices increase to 66 times that of the low carbon price scenario (Supplementary Table 2), the Mixed strategy achieves both the lowest total cost and CO_2 emissions (Supplementary Table 3). This implies that the Mixed strategy can only achieve the lowest CO_2 emissions among all battery strategies when carbon prices are extremely high, which is actually difficult to achieve in our real world.

Supplementary Table 1. Summary of national results for each battery storage deployment strategy assuming a 100-fold increase in low carbon prices and rapid battery costs decrease from 2025-2050

		RE-connected batteries	Grid-connected batteries	Demand-side batteries	Mixed batteries
Total cost (Billion dollars)		4088.6	4073.5	4337.1	4064.3
	Emission costs	65.2	63.1	112.5	61.1
	Gen fixed costs	3072.7	3050.9	3009.0	3049.3
	Gen variable costs	41.9	42.0	50.9	42.1
	Fuel costs	143.8	135.4	178.7	133.7
	Transmission costs	661.2	671.4	885.9	665.0
	Others	102.8	110.2	100.0	112.7
Cumulative CO_2 emissions (Mt)		34.3	33.2	59.0	32.1

Supplementary Table 2. The threshold of carbon prices (66 times that of the low carbon price scenario in Supplementary table 9) enables the Mixed battery strategy to achieve both the lowest total system costs and CO_2 emissions among the four battery strategies.

	2025	2030	2035	2040	2045	2050
carbon price (RMB/t CO_2)	4950	7920	10890	18480	28380	40260

*RMB represents Renminbi, which is the official currency of the People's Republic of China.

Supplementary Table 3. Summary of national results for each battery storage deployment strategy assuming a 66-fold increase in low carbon prices and rapid battery costs decrease from 2025-2050

		RE-connected batteries	Grid-connected batteries	Demand-side batteries	Mixed batteries
Total cost (Billion dollars)		4052.1	4039.7	4283.9	4031.3
	Emission costs	95.6	91.8	144.3	91.7
	Gen fixed costs	3019.6	3010.2	2938.9	3006.7
	Gen variable costs	42.4	42.2	52.3	42.1
	Fuel costs	196.5	185.9	242.4	182.2
	Transmission costs	655.9	664.8	855.8	661.1
	Others	42.1	44.8	50.2	47.4
Cumulative CO_2 emissions (Mt)		75.9	72.8	114.7	72.7

Supplemental Figures

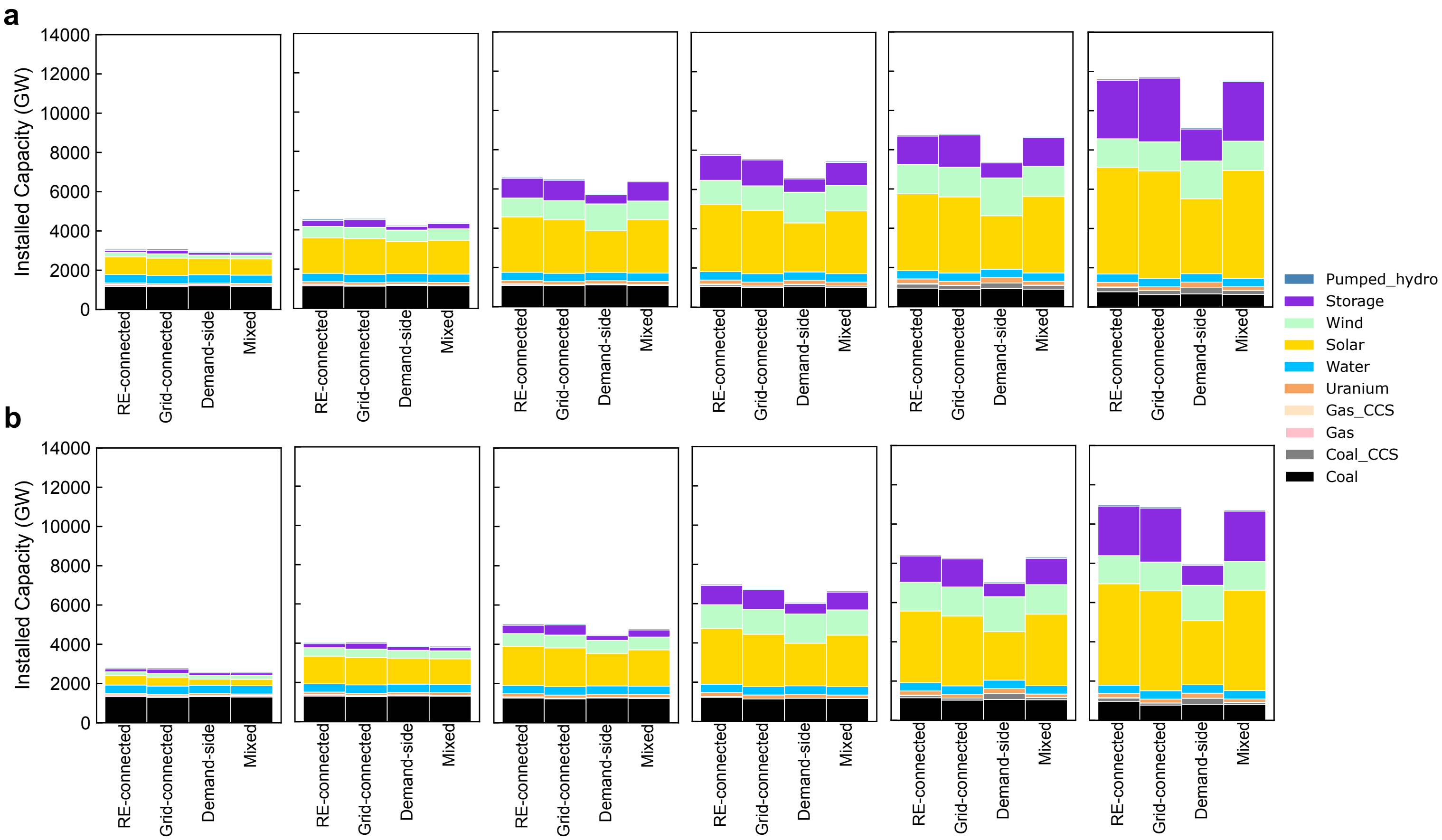

Supplementary Fig. 9. Optimal installed capacity of each generation technology from 2025-2050 under RE-connected, Grid-connected, Demand-side, and Mixed battery strategy with **a** Low carbon prices and **b** High carbon prices and rapid decreases in battery costs. Coal_CCS and Gas_CCS represent coal and gas power generation with carbon capture and storage.

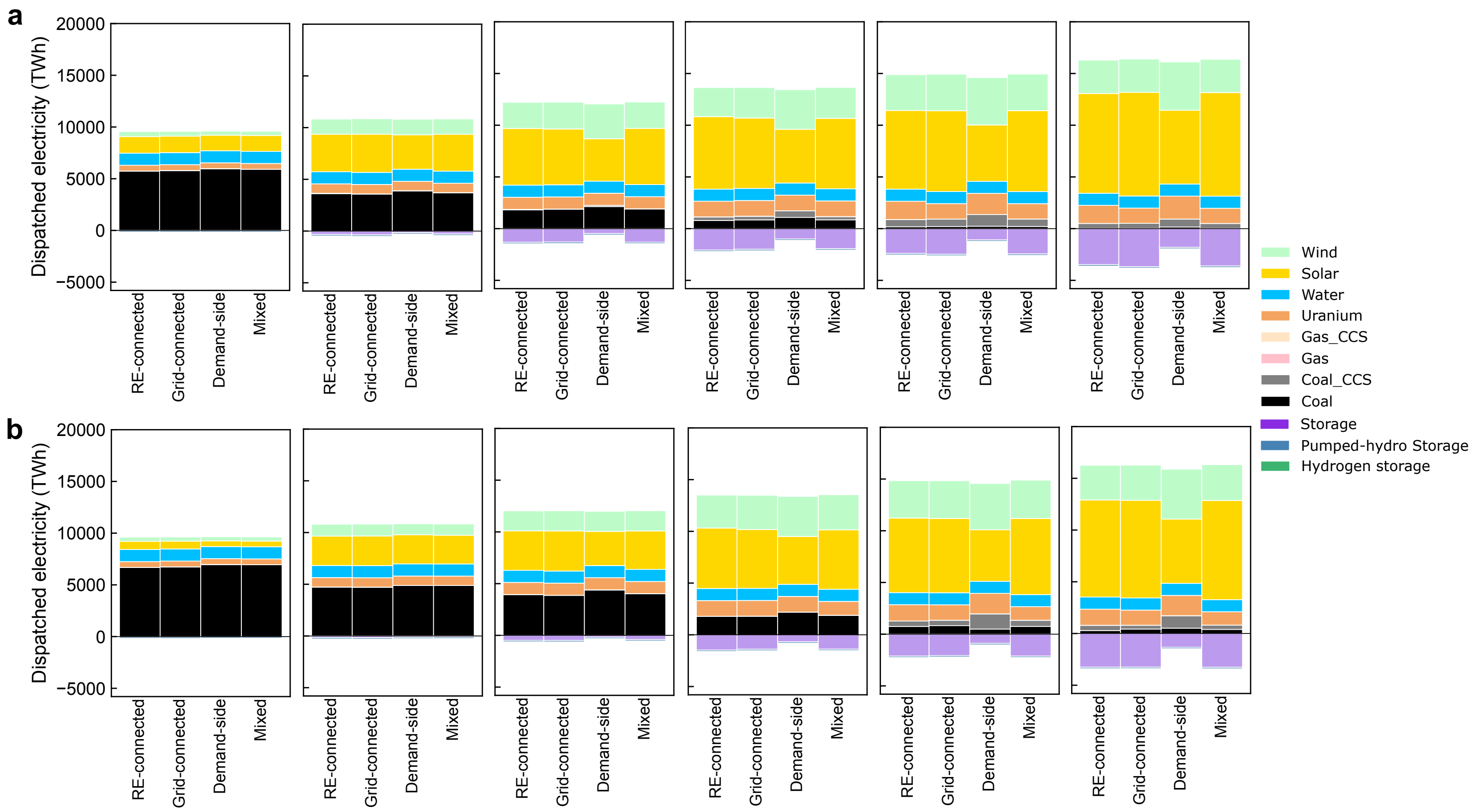

Supplementary Fig. 10. Optimal power generation of each generator technology and electricity discharged from energy storage from 2025-2050 under RE-connected, Grid-connected, Demand-side, and Mixed battery strategy with **a** Low carbon prices and **b** High carbon prices and rapid decreases in battery costs. Coal_CCS and Gas_CCS represent coal and gas power generation with carbon capture and storage.

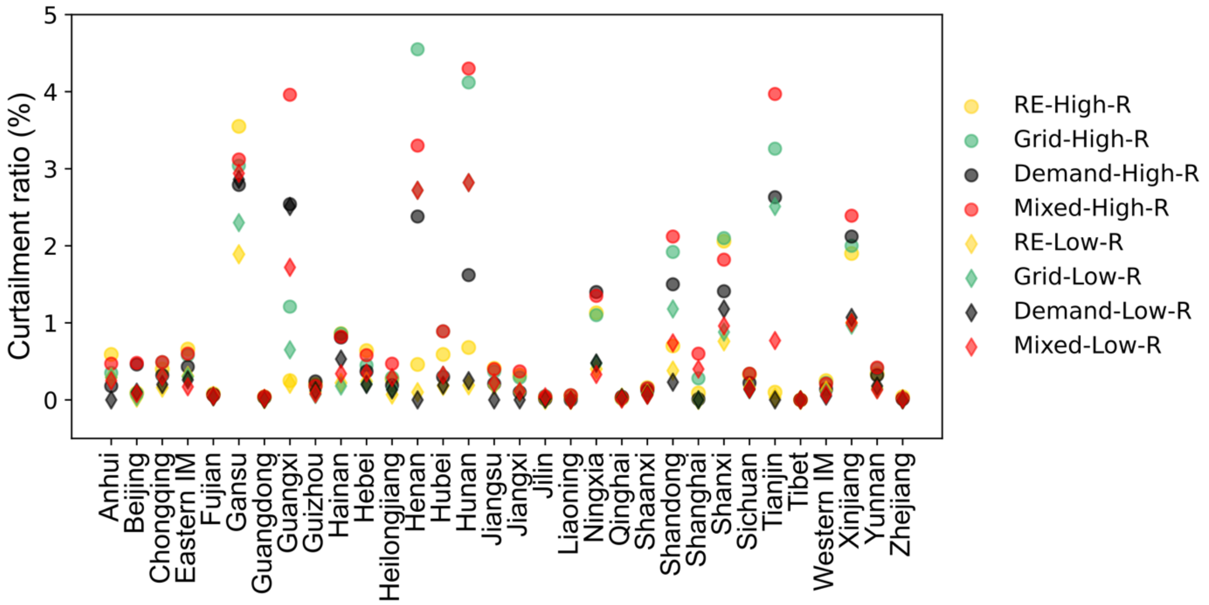

Supplementary Fig. 11. Comparison of average curtailment ratio of renewables (both solar PV and wind) in each province under eight scenarios. RE-High(Low)-R, Grid-High(Low)-R, Demand-High(Low)-R, and Mixed-High(Low)-R represent Renewable-connected, Grid-connected, Demand-side and Mixed battery strategy with high (low) carbon price and rapid decrease in battery costs.

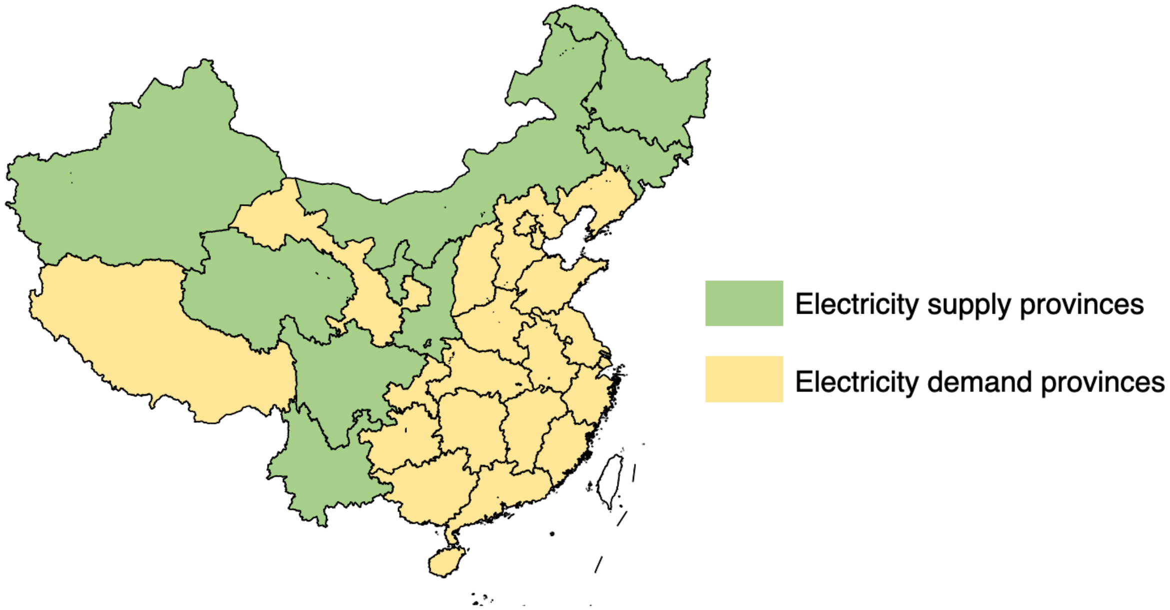

Supplementary Fig. 12. Distribution of electricity supply and demand provinces. The map of China is sourced from the National Catalogue Service for Geographic Information[1].

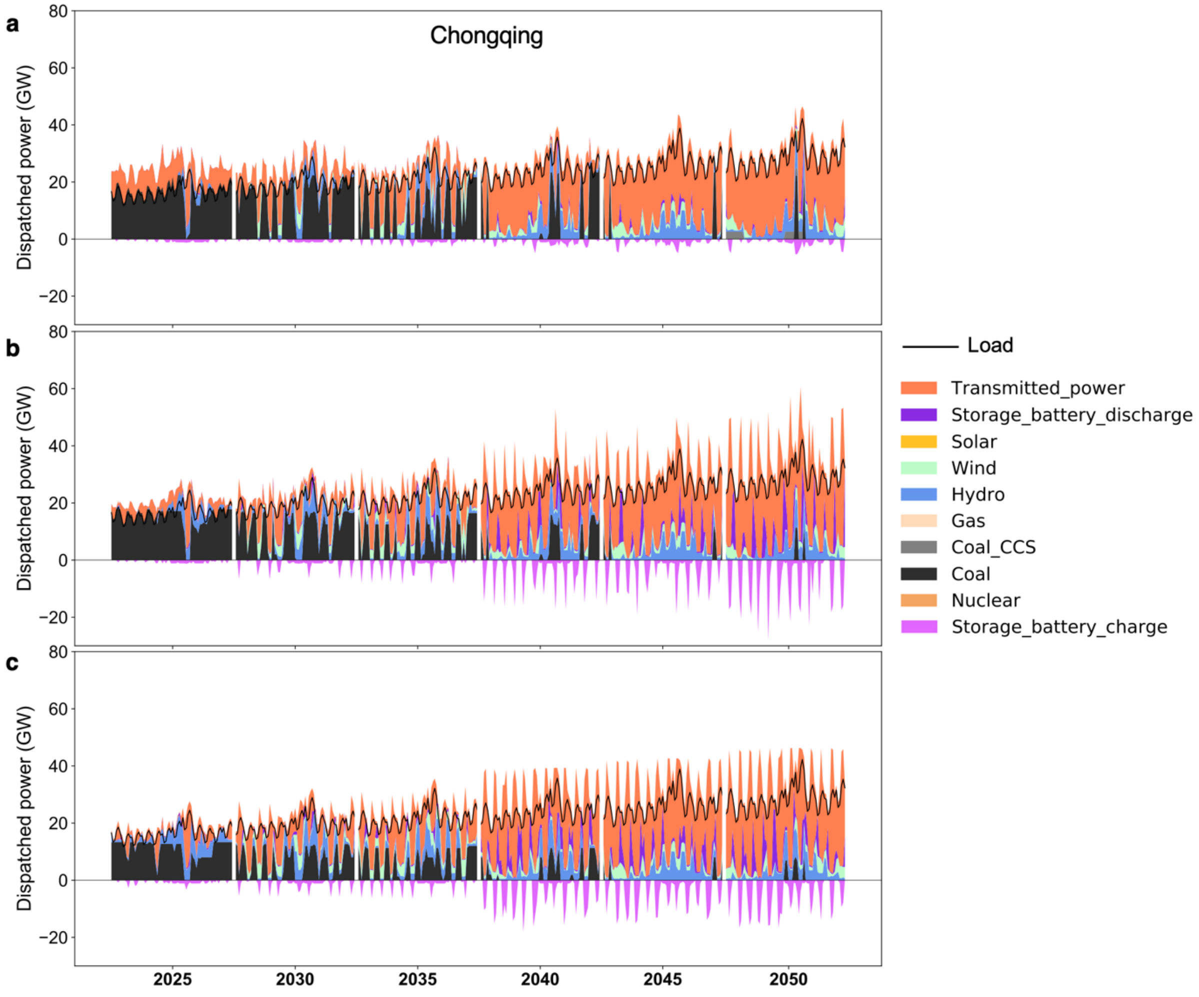

Supplementary Fig. 13. Dispatched power under three battery deployment strategies in Chongqing province

Dispatched power from various power generators, transmission, and battery discharges in **a** RE-connected battery, **b** Grid-connected battery and **c** Demand-side strategy with low carbon and rapid decreases in battery costs. Coal_CCS represents coal-fired power generation with carbon capture and storage.

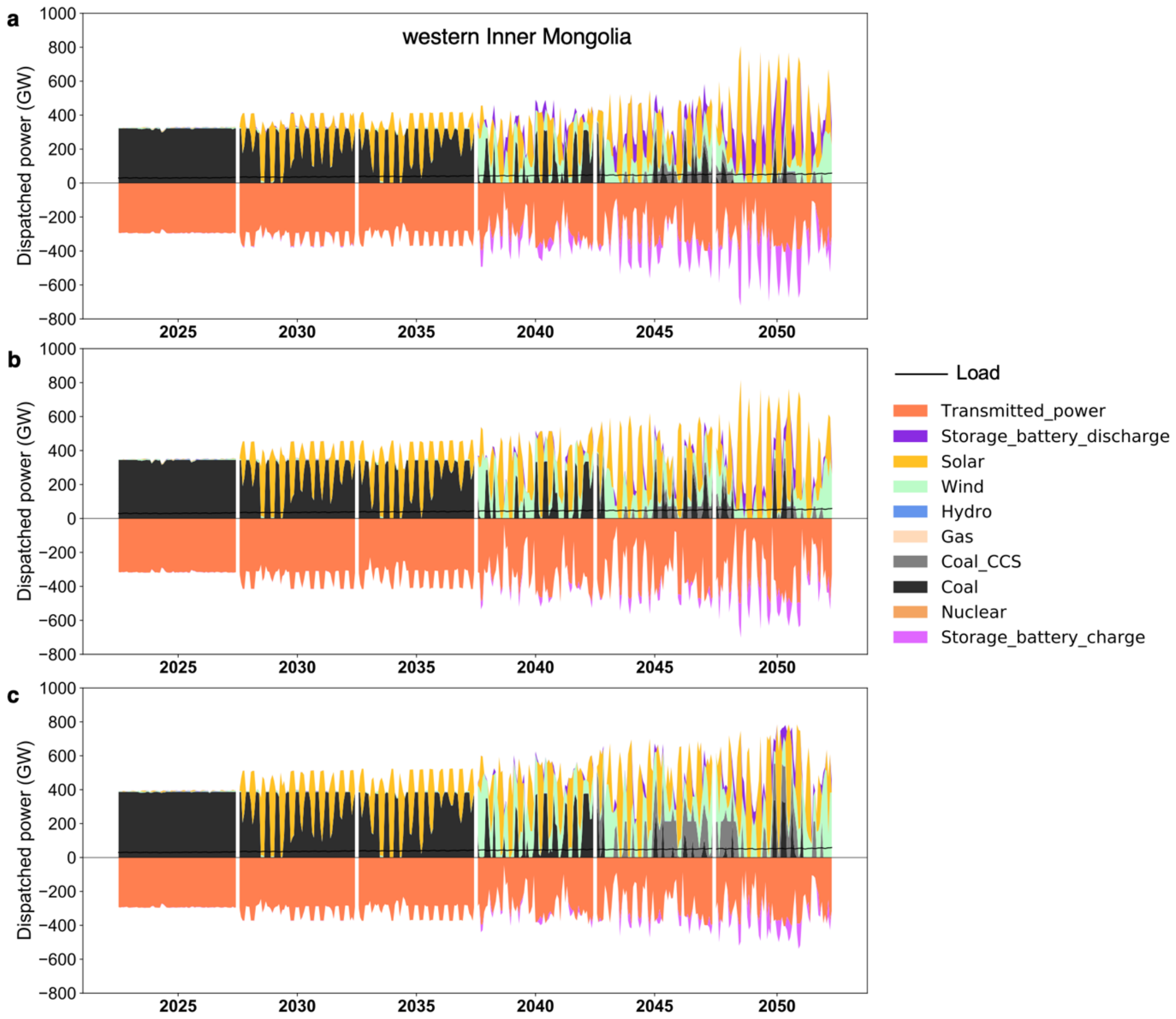

Supplementary Fig. 14. Dispatched power under three battery deployment strategies in western Inner Mongolia

Dispatched power from various power generators, transmission, and battery discharges in **a** RE-connected battery, **b** Grid-connected battery and **c** Demand-side strategy with low carbon and rapid decreases in battery costs. Coal_CCS represents coal-fired power generation with carbon capture and storage.

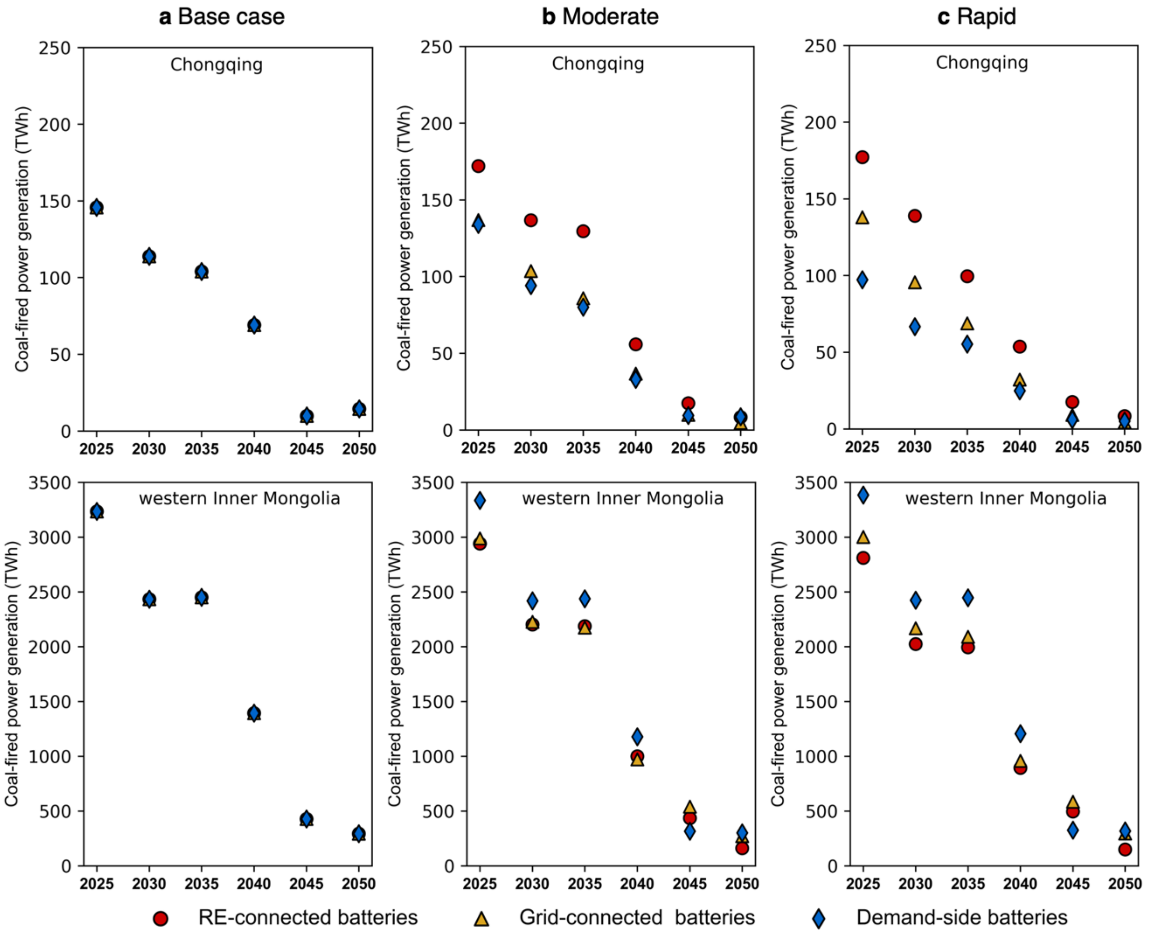

Supplementary Fig. 15. Comparison of provincial coal-fired power generation among three battery storage deployment strategies under low carbon prices with **a** base case, **b** moderate and **c** rapid battery cost decreases.

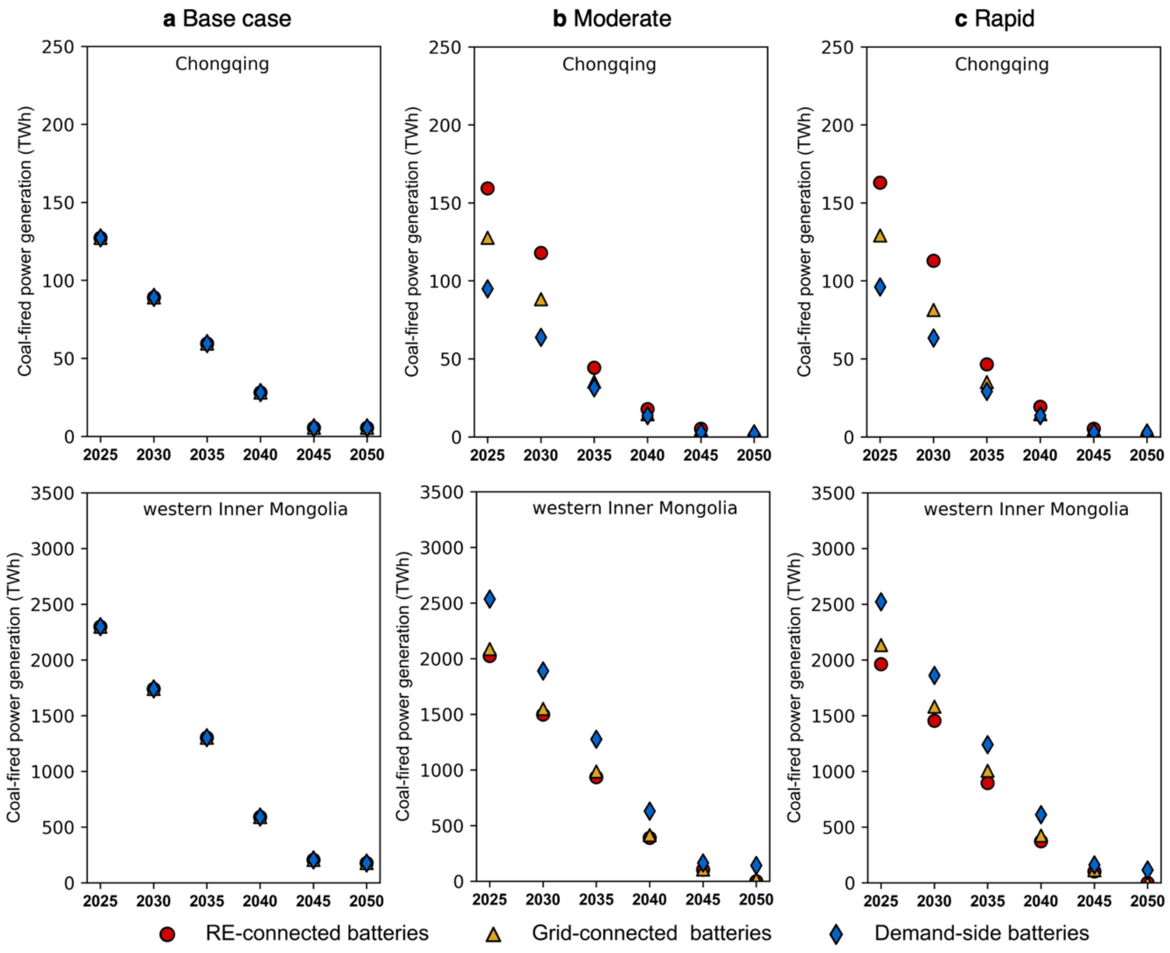

Supplementary Fig. 16. Comparison of provincial coal-fired power generation among three battery storage deployment strategies under high carbon prices with **a** base case, **b** moderate and **c** rapid battery cost decreases.

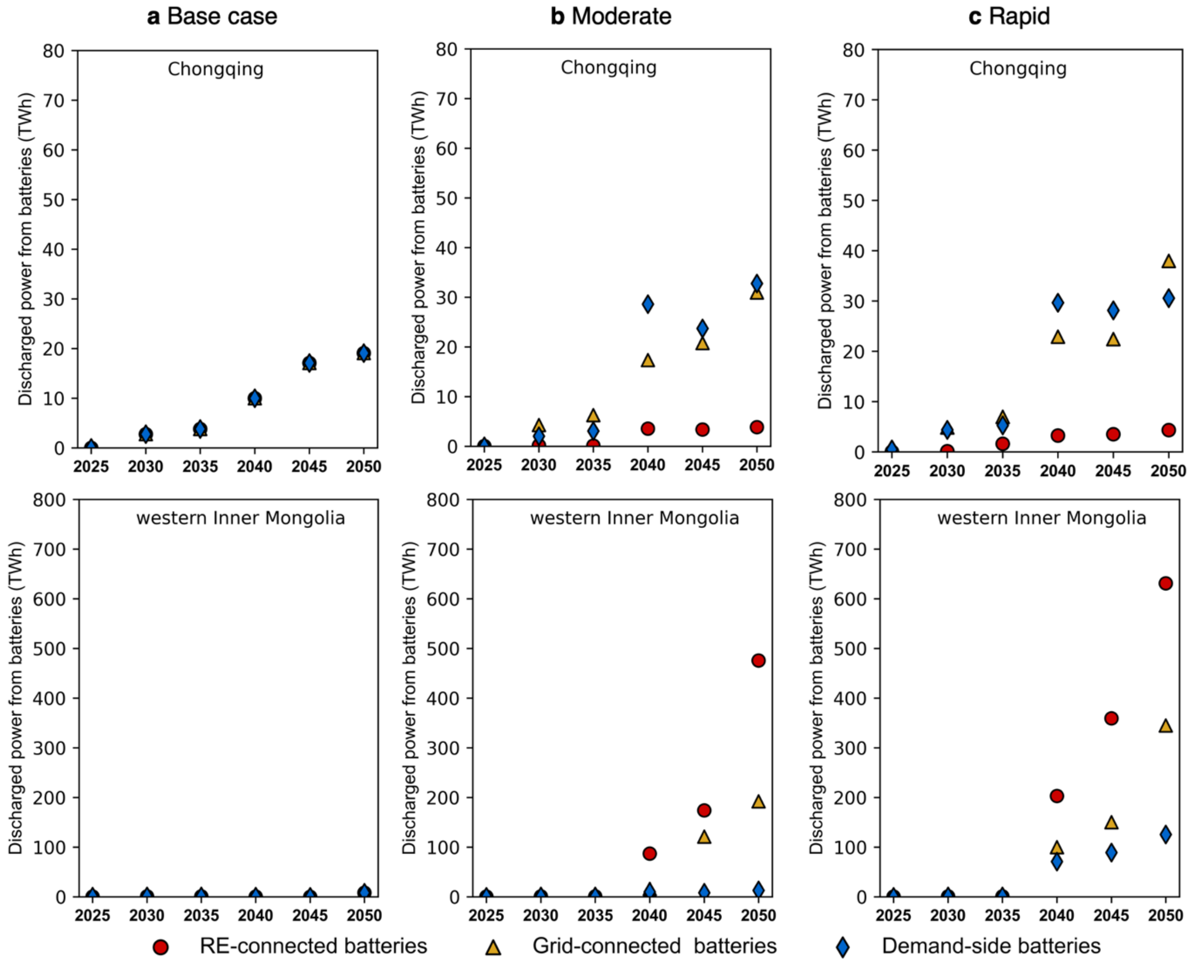

Supplementary Fig. 17. Comparison of provincial power discharged from batteries among three battery storage deployment strategies under low carbon prices with **a** base case, **b** moderate and **c** rapid battery cost decreases.

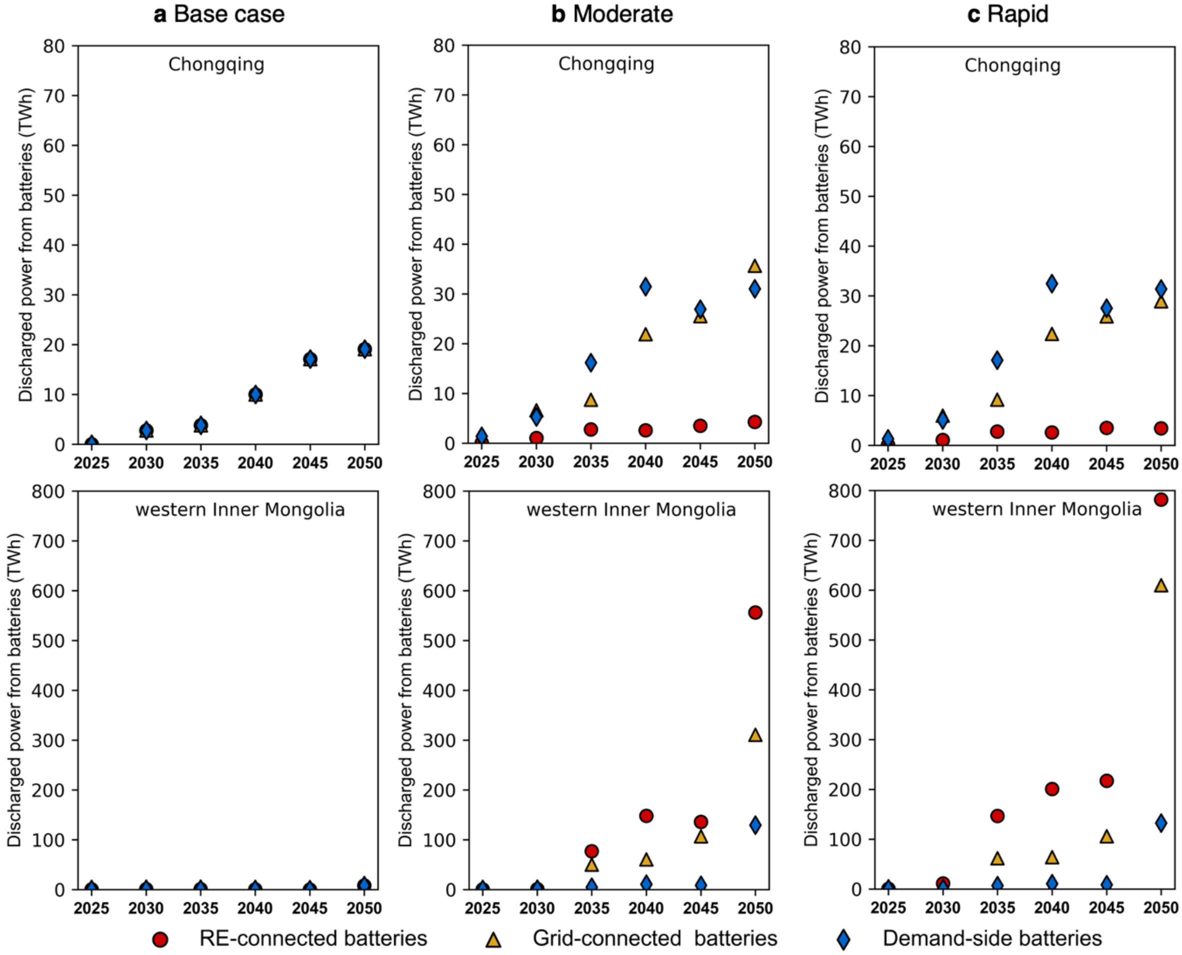

Supplementary Fig. 18. Comparison of provincial charged power for batteries among three battery storage deployment strategies under high carbon prices with three battery cost decrease **a** base case, **b** moderate and **c** rapid battery cost decreases.

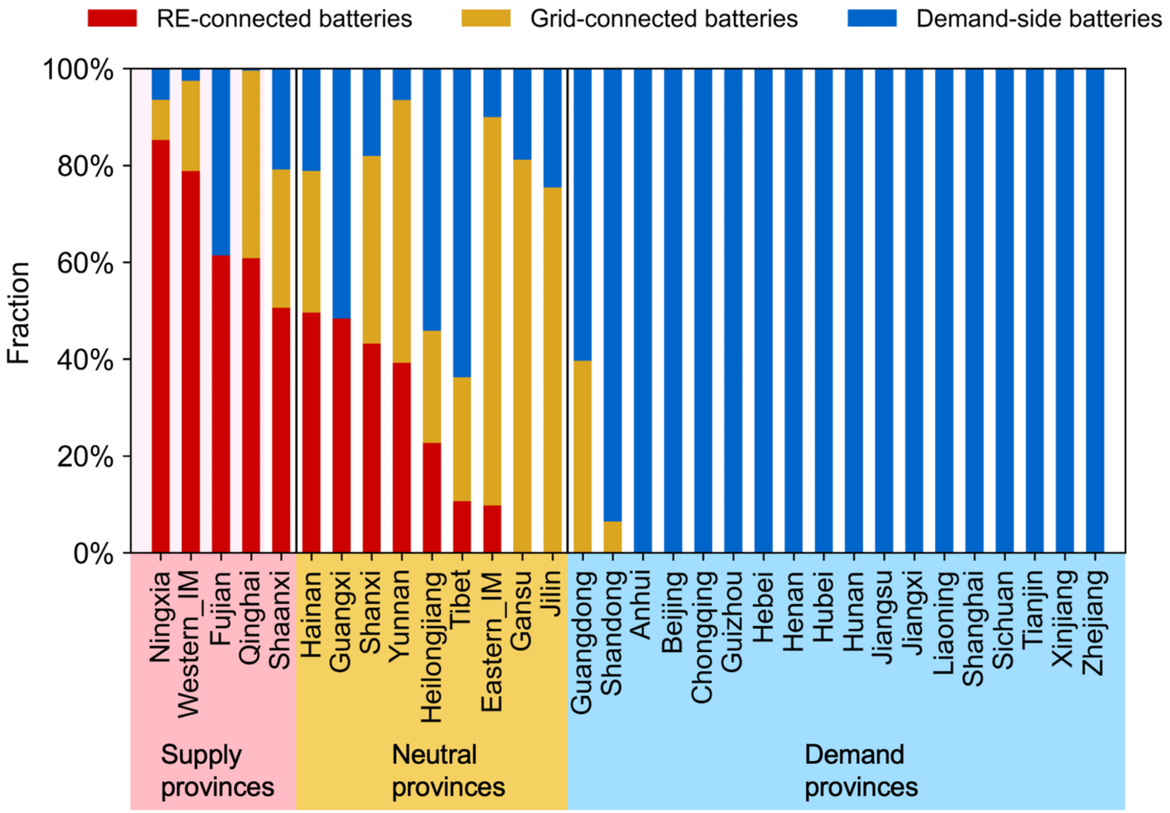

Supplementary Fig. 19. Battery storage deployment strategies used by each province in the Mixed-High-R scenario. Mixed-High-R represents Mixed battery strategy with high carbon price and rapid decrease of battery costs. Provinces in the pink shade are supply provinces, those in the yellow shade are neutral provinces, and those in the blue shade are demand provinces.

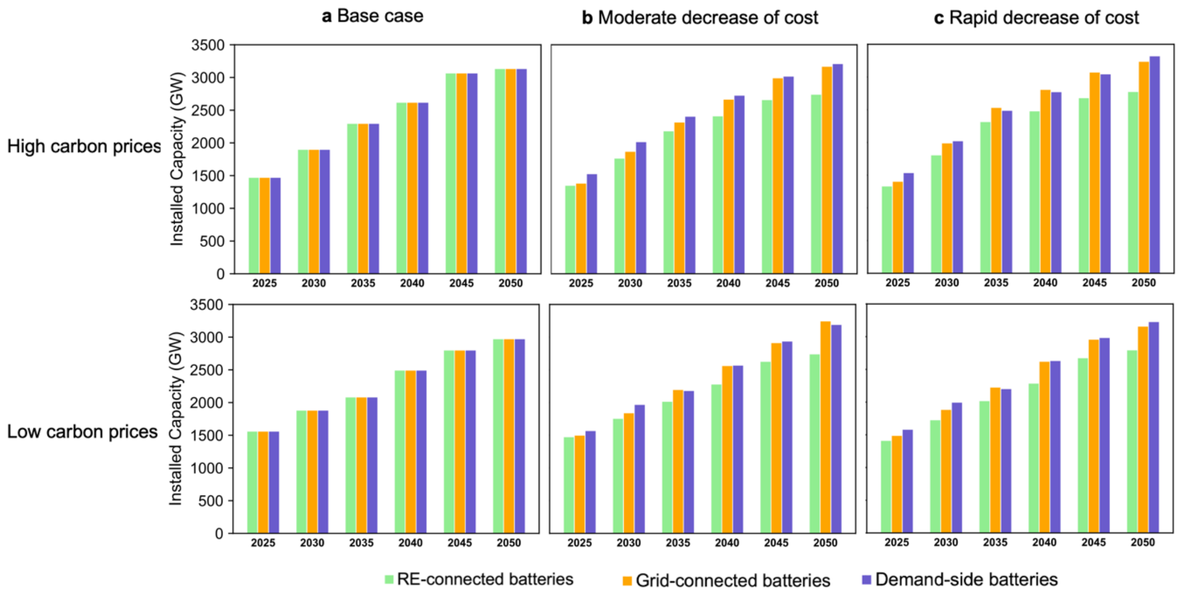

Supplementary Fig. 20. Transmission capacity of high and low carbon prices with **a** base case of battery and renewable costs, **b** moderate decrease of battery and renewable costs, and **c** rapid decrease of battery and renewable costs.

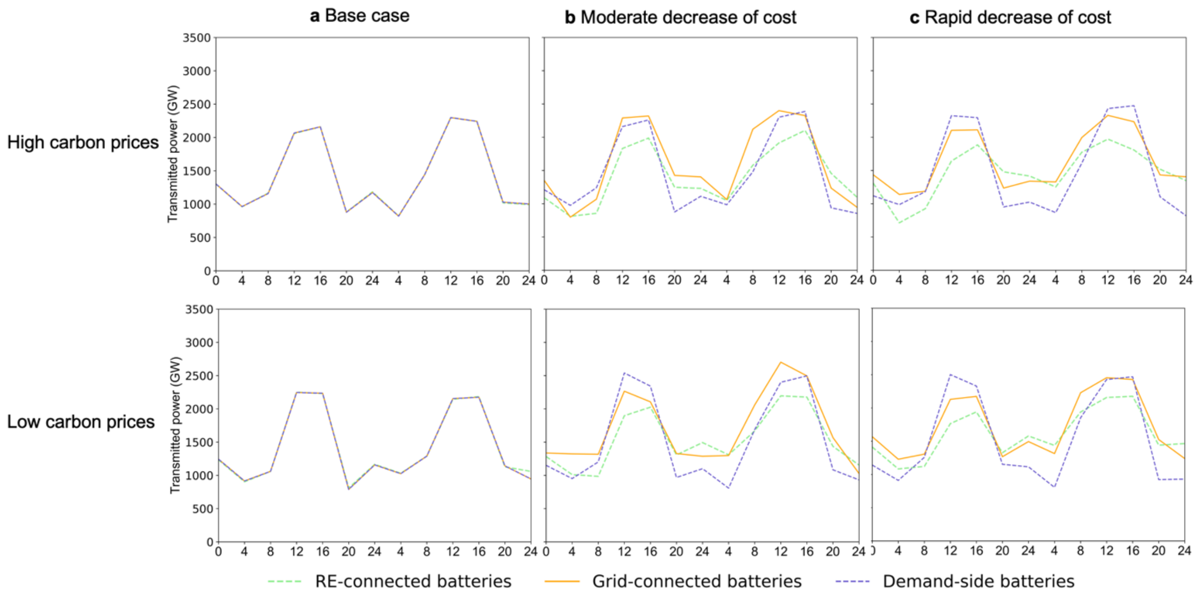

Supplementary Fig. 21. Transmitted power of high and low carbon prices with **a** base case of battery and renewable costs, **b** moderate decrease of battery and renewable costs, and **c** rapid decrease in battery and renewable costs.

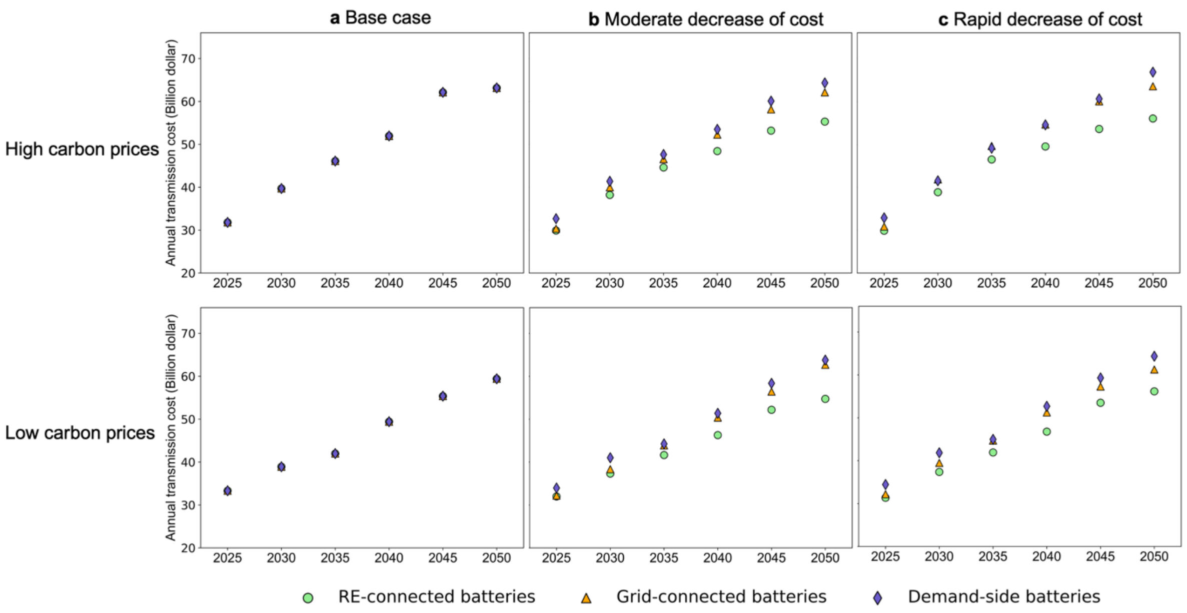

Supplementary Fig. 22. Annual transmission costs of high and low carbon prices with **a** base case of battery and renewable costs, **b** moderate decrease of battery and renewable costs, and **c** rapid decrease in battery and renewable costs.

Supplemental Tables

Supplementary Table 4. National average capital cost, fixed operation and maintenance costs and fuel costs of coal-fired, natural gas and nuclear power plants from 2020-2050 (BNEF, 2022, He et al., 2016).

Costs	Year	Coal	Gas	Nuclear
Capital costs ($/kW)	2020	598	532	2140
	2030	598	532	2140
	2040	598	532	2140
	2050	598	532	2140
Fixed operation and maintenance ($/kW)	2020	10.6	13.2	72
	2030	10.6	13.2	72
	2040	10.6	13.2	72
	2050	10.6	13.2	72
Fuel costs ($/MMBtu)	2020	4.5	13.7	0.82
	2030	4.8	15.6	0.90
	2040	5.3	16.9	1.06
	2050	5.8	16.9	1.22

Supplementary Table 5. Carbon emissions of each scenario (Unit: Tg). Here are the abbreviations and their meanings: Renewable-connected battery strategy (RE); Grid-connected battery strategy (Grid); Demand-side battery strategy (Demand); high carbon price (High); low carbon price (Low); base trajectory of battery costs (B); moderate trajectory of battery costs (M); rapid trajectory of battery costs (R).

Scenario	2025	2030	2035	2040	2045	2050
RE-Low-B	5644	4235	3750	1536	605	302
RE-Low-M	5084	3747	3336	1471	567	281
RE-Low-R	5143	3637	3023	1355	641	286
RE-High-B	4523	3097	1578	698	268	104
RE-High-M	4425	2964	1443	682	237	81
RE-High-R	4419	2803	1411	652	241	80
Grid-Low-B	5536	4112	3644	1700	698	321
Grid-Low-M	5064	3671	3176	1398	664	358
Grid-Low-R	5191	3626	2971	1377	696	374
Grid-High-B	4502	3011	1634	734	287	128
Grid-High-M	4408	2900	1452	717	249	98
Grid-High-R	4460	2766	1445	695	257	86
Demand-Low-B	5896	4334	3843	1878	835	498
Demand-Low-M	5293	3747	3392	1618	796	561
Demand-Low-R	5368	3751	3370	1668	582	559
Demand-High-B	4798	3109	1703	812	345	198
Demand-High-M	4617	3038	1716	937	350	257
Demand-High-R	4602	3002	1653	917	335	226

Supplementary Table 6. System costs of each scenario (Unit: Billion dollars). Here are the abbreviations and their meanings: Renewable-connected battery strategy (RE); Grid-connected battery strategy (Grid); Demand-side battery strategy (Demand); high carbon price (High); low carbon price (Low); base trajectory of battery costs (B); moderate trajectory of battery costs (M); rapid trajectory of battery costs (R).

Scenario	2025	2030	2035	2040	2045	2050
RE-Low-B	940	721	556	414	306	221
RE-Low-M	940	720	554	406	298	216
RE-Low-R	939	717	550	403	297	212
RE-High-B	1032	786	603	444	318	233
RE-High-M	1032	783	591	431	308	220
RE-High-R	1031	779	586	427	306	216
Grid-Low-B	939	721	556	414	306	221
Grid-Low-M	940	719	553	402	296	213
Grid-Low-R	936	716	548	398	294	209
Grid-High-B	1033	786	603	444	318	234
Grid-High-M	1033	782	591	427	305	218
Grid-High-R	1029	776	584	423	302	213
Demand-Low-B	939	721	556	414	306	221
Demand-Low-M	930	712	547	401	302	216
Demand-Low-R	927	709	545	399	301	215
Demand-High-B	1032	786	603	444	318	234
Demand-High-M	1026	776	591	431	312	227
Demand-High-R	1022	773	587	429	310	225

Supplementary Table 7. Summary of national results by each battery storage deployment strategy with high carbon prices and rapid battery costs decrease from 2025-2050

		RE-connected batteries	Grid-connected batteries	Demand-side batteries	Mixed batteries
Total cost (Billion dollars)		3344.4	3328.4	3347.7	3308.1
	Emission costs	367.5	371.4	411.7	378.8
	Gen fixed costs	1787.5	1765.4	1687.3	1733.5
	Gen variable costs	76.3	76.4	84.7	77
	Fuel costs	533.4	522.7	559.9	529
	Transmission costs	578.3	591.0	602.2	588.3
	Others	1.4	1.5	1.9	1.5
Cumulative emission (Mt)		9607.4	9710.2	10735.1	9903.1
Cumulative renewable capacities (GW)		24248.9	24007.3	21148.9	23912.4
Cumulative battery capacities (GW)		7128.8	7838.8	3857.2	7034.4

Supplementary Table 8. Summary of national results by each battery storage deployment strategy with low carbon prices and rapid battery costs decrease from 2025-2050

		RE-connected batteries	Grid-connected batteries	Demand-side batteries	Mixed batteries
Total cost (Billion dollars)		3118.9	3102.1	3197.6	3075.3
	Emission costs	269.6	272.4	292.9	280.1
	Gen fixed costs	1563.1	1540.4	1458.9	1494.3
	Gen variable costs	86.9	87.2	93.8	88.5
	Fuel costs	623.3	616.5	656.1	625.7
	Transmission costs	574.7	584.9	593.9	585.6
	Others	1.3	1.3	1.2	1.1
Cumulative emissions (Mt)		14104.7	14235.3	15301.4	14544.1
Cumulative renewable capacities (GW)		20921.7	20607.6	17513.3	20357.5
Cumulative battery capacities (GW)		5639.1	6253.7	4808.2	5493

Supplementary Table 9. Carbon price in the model

Scenarios (RMB/t CO_2)	2025	2030	2035	2040	2045	2050
Low carbon price	75[a]	120[a]	165[a]	280	430	610
High carbon price	150	240	330	550	860	1220

[a] CO_2 price is obtained from the projection by the State Grid Energy Research Institution (China Energy & Electricity Outlook 2019).

*CO_2 price in slow decarbonization scenario after 2035 is based on our own assumptions.

*CO_2 price in rapid decarbonization scenario is set twice the value of that in slow decarbonization scenario.

*RMB represents Renminbi, which is the official currency of the People's Republic of China.

Reference:

1. National Catalogue Service for Geographic Information. GlobeLand30. https://www.webmap.cn/mapDataAction.do?method=globalLandCover.
2. NREL. *National Renewable Energy Laboratory. Annual Technology Baseline (ATB).* https://atb.nrel.gov/electricity/2021/data (2021).
3. He, G. *et al.* SWITCH-China: A Systems Approach to Decarbonizing China's Power System. *Environ. Sci. Technol.* **50**, 5467–5473 (2016).
